# Evidence-based medicine among physicians working in selected public hospitals in eastern Ethiopia: a cross-sectional study

**DOI:** 10.1186/s12911-019-0826-8

**Published:** 2019-06-03

**Authors:** Teshager Worku, Meron Yeshitila, Tilaye Feto, Shiferaw Leta, Frehiwot Mesfin, Haymanot Mezmur

**Affiliations:** College of Health and Medical Sciences, Haramaya University, PO. Box 235, Harar, Ethiopia

**Keywords:** Evidence-based medicine, Public hospitals, Physicians, Eastern Ethiopia, Barriers

## Abstract

**Background:**

Evidence-Based Medicine (EBM) is the process of systematically locating, searching, evaluating, and using contemporaneous research findings as the basis for clinical decision making. The systematic review showed that there is a considerable gap between what is known in the systematic research evidence and what happens in practice. Thus, the purpose of this study is to assess knowledge, attitude, practice and perceived barriers to EBM among physicians working in public hospitals in eastern Ethiopia.

**Methods:**

An institutional-based cross-sectional survey was conducted from April 1–June 8, 2017. Simple random sampling with proportional allocation was used. A total of 137 physicians was included in the survey. The data were collected by interview. Data were coded and entered to EpiData 3.1 then exported to and analyzed by using IBM SPSS statistics 21.0.

**Results:**

Physicians were aware of and used HINARI (22.6%), Cochrane (29.8%) and PubMed/Medline (37.9%) EBM electronic databases. The majority, (88.7%) physicians have a good attitude to EBM but only (32.3%) integrate it into clinical practice. Ability to retrieving evidence, evaluating the outcomes of the EBM practice implemented and difficulty in understanding research reports were significantly associated factors.

**Conclusions:**

The attitude of the physicians towards EBM was virtuous, but knowledge of EBM and practice of integrating new evidence in healthcare service were really insufficient. Relatively, the EBM implementation is low when compared with many studies. To obviate this, the stakeholders need to have a strong commitment to design a strategy for promoting physicians in implementing EBM to their day to day clinical decision-making process.

## Background

Evidence-Based Medicine (EBM) is “the process of systematically locating, searching, evaluating, and using contemporaneous research findings as the basis for clinical decisions making” [1]. The practice of EBM means using the best clinical evidence from systematic research of specific clinical problems by combining with individual clinical expertise and client choice for clinical decision making. It involves the consideration of three elements: 1) valid and reliable research and current best evidence related to specific clinical questions, 2) clinical expertise of the practitioner, and 3) values and preferences of the patient/family [2–4]. Therefore, EBM has been considered as a cornerstone to improving the quality of health services and achieving quality in patient care [5].

Evidence-based practice implementation helps in efficient use of resources, decreasing costs, increasing patient satisfaction, and improving patient care [6]. In a study that was conducted in the USA shows that clients’ outcomes are at least improved by 28% when clinical care is based on best evidence rather than the use of evidence-based practice guidelines [7].

The systematic review showed that, despite EBM significance, its implementation in hospitals is a difficult activity. The major barriers to the practice of EBM were related to inadequate knowledge, and skill related to EBM, patient overload and lack of personal time [3]. Similarly, in relatively low and middle income countries, attitude, understanding, lack of resources, poor access to information resources, lack of knowledge and financial shortage [8, 9], lack of staff experienced in the use of EBM, insufficient administrative support, and limited access to information are the most common barriers to the implementation of EBM [10–12]. To identify the implementation of EBM, it is critical to assess knowledge, attitude, practice, and perceived barriers because it affects EBM implementation [9].

Despite the fact EBM is supported by numerous people, it is criticized by people, mostly come from within the medical professions. Their argument is, “In addition to the many scientific problems of creating sound guidelines when the evidence is weak, they stress the destructive effects of standards at the local level. Instead of using clinical judgment, practitioners will be encouraged to follow protocols that treat all patients as essentially interchangeable. Providers will therefore be poorly equipped to contend with the variations between patients they will encounter in actual clinical circumstances. Even more problematically, traditional health care professionals may be replaced by less expensive, less skilled workers, who may be incapable of operating effectively in diverse situations” [13].

Little is known about the knowledge, attitudes, awareness, practices and associated factors regarding EBM in Ethiopia, only a few studies were conducted in Addis Ababa, the capital of Ethiopia [12, 14]. But, as far as this search is concerned there was no found research conducted on EBM in Eastern Ethiopia. Therefore, Information obtained from this study will help in motivating hospitals and health Bureaus in developing different strategies for enhancing the practice of EBM.

## Methods

### The aim of the study

The study aim at assessing the Knowledge, Attitude, Practice and perceived barriers/challenges in EBM implementation among physicians (General practitioners, specialists, dentists or any medical practitioners) working in public hospitals in Eastern Ethiopia.

### Study area and period

The study was conducted at selected public hospitals in Eastern Ethiopia, specifically in Harari regional state, Dire Dawa administration and East Hararge Zone, from April 1–June 8, 2017. Harar is the capital of Harari regional state, located 515 km away from Addis Ababa. It has 2 public, 2 military and 2 private hospitals [15]. Dire Dawa city administration is 515 KM away from Addis Ababa. Dire Dawa is center of Dire Dawa administration and the second largest city in Ethiopia. It has two public Hospitals and 16 Health centers [16].

East Hararghe is one of the zones of the Oromia regions. It is bordered on the Southwest by the Shebelle River which separates it from the Bale, on the West by West Hararghe, on the North by Dire Dawa and on the North and East by the Somali region. It has 4 Hospitals and 83 Health centers [16].

The source of information /EBM resources/ for Eastern Ethiopia physicians is medical journals, thesis papers, electronic databases, internet, conferences, seminars, Government publications, reports, statistics, books, and guidelines. Recently, internet-based EBM information sources are becoming the dominant and the main resources. The wireless internet is the commonest types of internet service found in many hospitals. Although the internet is a key source of EBM information, the internet infrastructure in most of the hospitals is poor. The computer is not supplied by hospitals, it is fulfilled by the physicians themselves. So, the main source of internet is their mobile phone which they bear the cost for the internet service. In most of the cases printed information sources are distributed and delivered to the hospitals and physicians, but sometimes may not be distributed timely [17].

### Study design and participants

The facility-based cross-sectional study design was employed. Physicians with or without specialty and dentists working in selected public hospitals in Eastern Ethiopia who worked at least for one year after graduation were participants of the study. But physicians who were sick, not present during the period of data collection and not agree to participate in the study were excluded.

### Sample size determination and sampling technique

The sample size was calculated using single population proportion formula n = (Z α/2) ^2^ p (1-p) /d^2^; where, n = sample size, Zα/2 (1.96): significance level at α =0.05, P: expected proportion for Evidence-Based Medicine in Ethiopia *P* = 10% (0.1) [12], and d: margin of error (0.05). The calculated total sample size was 124 with 10% nonresponse rate, the final sample size was 137.

**Fig. 1:**
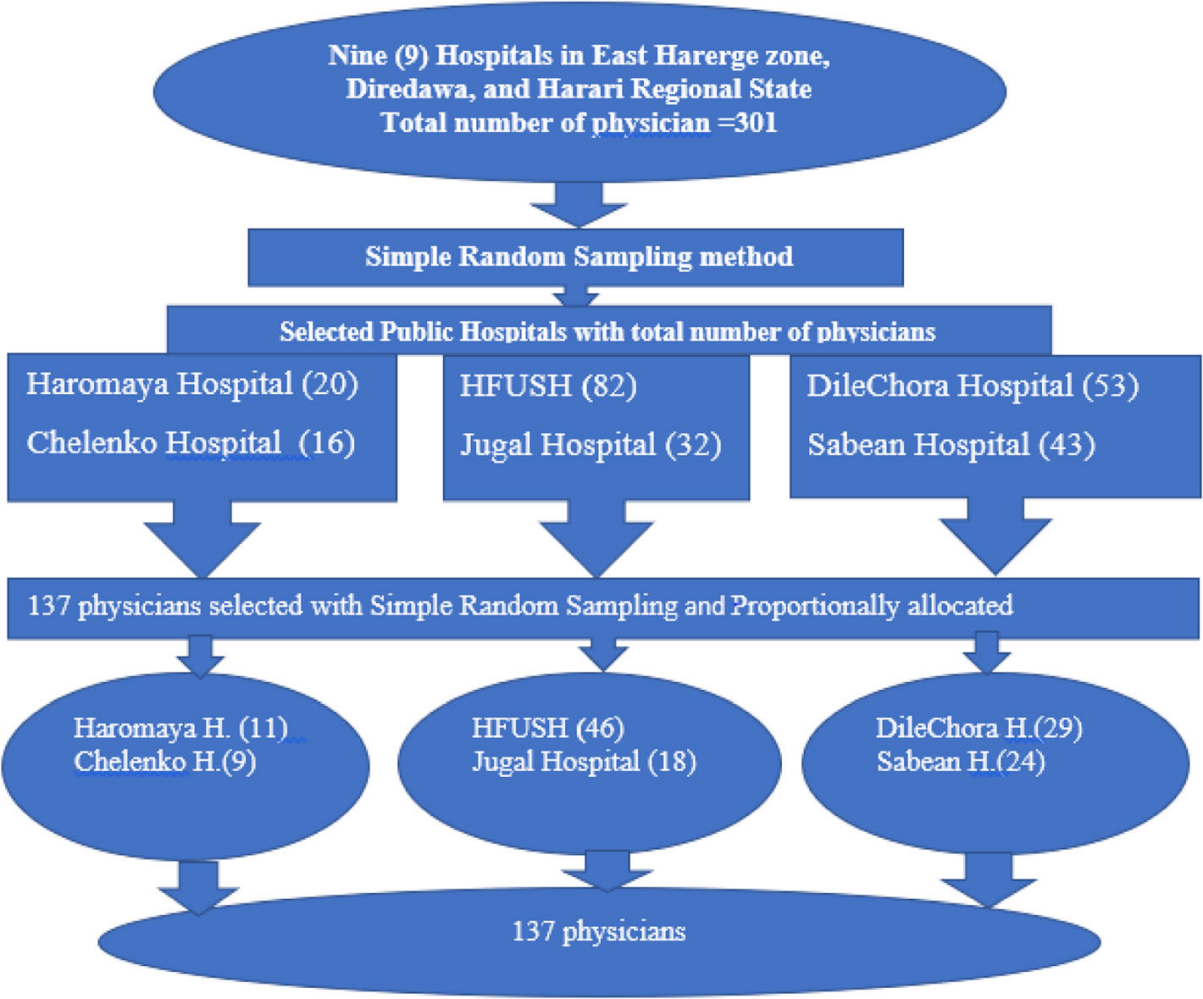
Schematic representation of the sampling procedure of selected Six Public Hospitals in eastern Ethiopia

In the East Hararghe zone, Dire Dawa Administration, and Harari Regional State, there are nine Public Hospitals, Deder, Haromaya, Bisidimo, Chelenko, Dilechora, Sabean, Jugal, Grawa Hospitals, and Hiwot Fana Specialized University Hospital. From these all Hospitals, we choose six public Hospitals by simple random sampling method. The selected Hospitals were Chelenko, Sabean, Jugal, Haromaya, Dilechora Hospitals and Hiwot Fana Specialized University Hospital. By taking the lists of physicians from the Human Resources of each six selected Hospitals, we determined the proportionate sample to be taken, to proportionate number of study subjects for each hospital, the formula = *(n)x(nf)/N* was used where n = number physicians in each Hospital, nf = total sample size and N = the total number of physicians in the six Hospitals. Study participants were by using a simple random sampling method that was 137 physicians. The distribution was allocated proportionally to the Six Hospitals (Fig. 1).

### Measurements and data collection techniques

A five-part questionnaire was adapted from two questionnaires from the available literature, The McColl and the BARRIERS scale questionnaires [18–21]. Part one contains personal and professional characteristics of physicians; Age, gender, unit of work, graduate level, number of years of practice and others. Part two covers physician’s sources of knowledge for current practice (EBM information resource, EBM technical terms, English Language skills, and Internet utilization skill, Involvement in research and development projects). Part three comprises the attitudes of physicians. Part four encompasses the practice of EBM and Part five embraces perceived barriers to EBM.

Physicians were selected based on the inclusion criteria. The data collectors interviewed the Physicians individually as one-on-one in a calm, relaxing and confidential space. The data collectors filled the questionnaires during the interview with the physicians. The incomplete response was excluded. Data was collected by a total of six BSc Degree Holder Nurses.

### Data quality assurance and management

The data collectors and supervisors were trained prior to the actual data collection about the purpose of the study, sampling procedure, methods of data collection, ethical issues, and ways of addressing contingency management. The questionnaire was pre-tested on 5% of the similar population at Bisidimo Hospital for consistency of response and validity of the questionnaire. Some adjustments were made while adapting the questionnaire to the Ethiopian context (some terminology were explained). Some concepts were replaced by the familiar concepts in the area). The supervisors and principal investigators closely followed the data collection process and ensured completeness and consistency. The incomplete questionnaires and those that seem like fake and not genuine (incoherent and contradectery ressponses) were excluded.

### Data processing and analysis

After the interview, data were checked manually for comprehensiveness and uniformity of responses. The data were coded and entered into Epi Data 3.1 then exported to and analyzed by using IBM SPSS statistics 21.0. The data were filtered by running descriptive statistics to explore some data glitches. The descriptive statistical analysis was applied to compute the mean standard deviations frequency and percentages, Binary, and multiple logistic regressions were conducted to examine the relationship between knowledge, attitude & practice of physicians to EBM & other variables. All analysis was carried at the 0.05 significance level and 95% CI.

## Results

### Socio-demographic characteristics

A total of 124 participated in the study with a response rate of (90.5%). The non- response rate included 10 none agree participants; three fake and seems not genuine data which were excluded. The majority of the participants were male physicians, namely 88 (71%). The mean age of the respondents was 29 ± 6.08 years and 117 (94.4%) were not having any managerial position. Most of the physicians were general practitioners which account for 110 (88.7%) and 2 (1.6%) were dentists. The majority, namely 90 (72.6%) participants have a work experience of 1–5 years. Near to quarter of physicians, 29 (23.4%) were at work in the surgical ward (Table 1).

**Table 1:** Socio-demographic characteristics of physicians in Eastern Ethiopia, June 2017. (*n* = 124)

Variables	Categories	Frequency	Percentage
Sex	Male	88	71
	Female	36	29
Level of Education	GP	110	88.7
	Specialist	12	9.7
	Others	2	1.6
Current unit of work	Medical ward	24	19.4
	Pediatrics	15	12.1
	Surgical ward	29	23.4
	Emergency	14	11.3
	Obs/Gyn	17	13.7
	OR	12	9.7
	Others	13	10.7
Working experience	1-5 yr.	90	72.6
	6-10 yr.	18	14.5
	11-15 yr.	9	7.3
	16-20 yr.	4	3.2
	21-34 yr	3	2.4
Have Position	Yes	117	94.4
	No	7	5.6

### Knowledge and awareness of EBM

From the total of 124 Physicians, most of the participants, namely 79 (63.7%) have all of the English language skill that is necessary to review articles written in English. In addition, 66 (53.2%) of the participants can effectively search for scientific articles (evidence) from the internet and 64 (51.6%) were involved in research and development projects.

### Knowledge of EBM resources

**Fig. 2:**
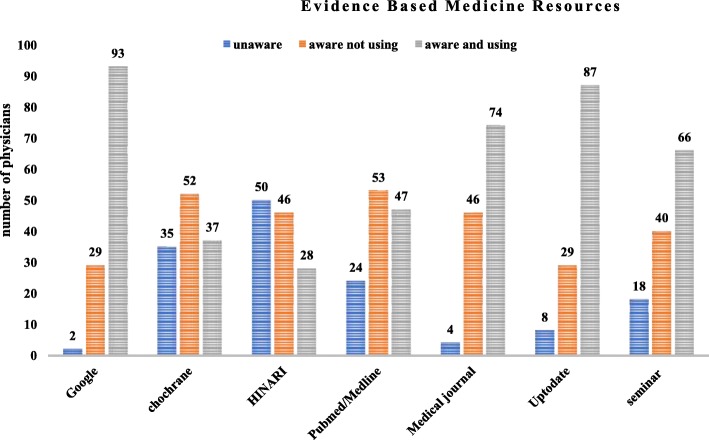
Knowledge of EBM resources of physicians in Eastern Ethiopia, June 2017. (*n* = 124)

Physicians were aware of and used HINARI 28(22.6%), Cochrane 37(29.8%) and PubMed/Medline 47 (37.9%) EBM electronic Databases. Despite this, Google and UpToDate were well understood and utilized by physicians 93 (75%) and 87 (70.2%) respectively (Fig. 2).

### Understanding of EBM technical terms

Only some of the physicians had an understanding and were able to explain to others the technical terms used in EBM such as confidence interval 55 (44.4%), relative risk (the ratio of the probability of an outcome in an exposed group to the probability of an outcome in an unexposed group) 41 (33.1%) the absolute risk (probability or chance of an event) 25 (20.2%) odds ratio (a statistic that quantifies the strength of the association between two events) 24 (19.4%), and others (Table 2).

**Table 2:** Understanding of EBM research, technical terms of physicians in Eastern Ethiopia, June 2017. (n = 124)

Variables	Don’t understand	Some understanding	Understand and could explain to others
Odds Ratio	37 (29.8%)	63 (50.8%)	24 (19.4%)
Relative Risk	24 (19.4%)	59 (47.6%)	41 (33.1%)
Absolute Risk	32 (25.8%)	67 (54%)	25 (20.2%)
Systematic Review	36 (29%)	60 (48.4%)	28 (22.6%)
Meta- Analysis	34 (27.4%)	57 (46%)	33 (26.6%)
Clinical Effectiveness	35 (28.2%)	60 (48.4%)	29 (23.4%)
Confidence Interval	21 (16.9%)	48 (38.7%)	55 (44.4%)
Publication Bias	34 (27.4%)	58 (46.8%)	32 (25.8%)

### Physician’s attitudes and level of use of evidence-based medicine

In our finding nearly all, 110 (88.7%) physicians have had a good attitude to EBM. Among the total participants, only 40 (32.3%) integrated EBM in their clinical practice. A few physicians practiced EBM in their day to day practice through Identifying knowledge gaps 7 (5.6%), formulate answerable questions 3 (2.4%), locating and searching the relevant evidence 3 (2.4%), retrieving evidence 19 (15.3%), critically appraised evidence 3 (2.4%), evaluate the outcomes of EBM implementation 15 (12%) and share information with colleagues 29 (23.4%).

### Perceived barriers in EBM among physicians

#### Barriers to finding and reviewing research and changing practice

According to this finding, the most important barriers to physicians in finding and reviewing research findings is that organization doesn’t have access to non-free electronic databases 77 (62.1%). Next, to this, they don’t know how to find research reports 66 (53.2%) followed by insufficient time and lack of interest to find research reports 64 (51.7%). The English Language seems not to be a problem for physicians in searching and retrieving research evidence 120 (96.8%). Similarly, the main barriers which were regarded as obstacle to implementation of EBM despite available evidence, were insufficient resources (e.g. Equipment, internet availability) to change own practice 95 (76.6%), insufficient time at work to implement changes in own practice 72 (58.1%) and team culture was not receptive to changing practice 72 (58.1%), (Table 3).

**Table 3:** Barriers to finding and reviewing research and barriers to changing practice among Eastern Ethiopia Physicians, June 2017, (n = 124)

Variables	No	Yes
***Barriers to finding and reviewing research***		
Incompetency in English Language	120 (96.8%)	4 (3.2%)
Computer illiteracy	70 (56.5%)	54 (43.5%)
Research reports not easy to find	61 (49.2%)	63 (50.8%)
Insufficient time/lack of interest to find research reports	60 (48.3%)	64 (51.7%)
Lack of confidence in judging the quality of researches	77 (63%)	47 (37%)
Difficulty in understanding research reports	78 (62.9%)	46 (37.1%)
Don’t know how to find research reports	58 (46.8%)	66 (53.2%)
Organizational data base not easy to find	47 (37.9%)	77 (62.1%)
***Barriers to changing practice***		
Insufficient resources (e.g. equipment, internet) to change own practice	29 (23.4%)	95 (76.6%)
Insufficient time at work to implement changes in own practice	52 (41.9%)	72 (58.1%)
Team culture is not receptive to changing practice	52 (41.9%)	72 (58.1%)
Lacking the authority to change practice	57 (46%)	67 (54%)
Lack of confidence about beginning to change own practice	56 (45.2%)	68 (54.8%)

#### Factors associated with the practice of EBM among physicians

Difficulty in understanding research reports, ability to retrieving evidence and evaluating the outcomes of EBM were significantly associated with the implementations of EBM in clinical practice. Physicians who had difficulty in understanding research reports were 30% times less likely to implement EBM into practice as compared to those who do not have difficulty in understanding research reports (AOR = 0.72,95% CI: 0.525–0.993, *P* = 0.045). Those physicians who reported that they are able to retrieving evidence were 3.15 times more likely to implement the EBM when compared to those who are unable to retrieving evidence well (AOR = 3.15, 95% CI: 1.10–9.01, *P* = 0.03). Those physicians who evaluated the outcomes of EBM implemented were 3.45 times more likely to implement EBM more frequently than those who did not evaluate the outcomes of EBM implemented (AOR = 3.45, 95% CI: 1.23–9.67, P = 0.03), (Table 4).

**Table 4:** The association between independent variables and EBM among physicians in Eastern Ethiopia, June 2017 (n = 124)

Evidence-Based Practice Integration	Yes	No	COR (95% CI)	*P*-value	AOR (95% CI)	*P*-value
	40 (32.3)	84 (67.7)				
Aware and Use PubMed/Medline	47 (37.9%)	77 (62.1%)	1.864 (1.073–3.236)	0.027	1.067 (0.395–2.884)	0.89
Aware and Use UpToDate	87 (70.2%)	37 (29.8%)	2.613 (1.152–5.926)	0.022	2.037 (0.604–6.868)	0.251
Aware and Use Absolute Risk	25 (20.2%)	99 (79.8%)	1.824 (1.022–3.254)	0.042	0.660 (0.219–1.991)	0.461
Able to Retrieving evidence	60 (48.4%)	64 (51.6%)	1.770 (1.186–2.643)	0.005	3.149 (1.100–9.012)	0.032*
Critically appraised any literature discovered	44 (35.5%)	80 (64.5%)	1.685 (1.139–2.495)	0.009	0.9669 (0.354–2.634)	0.946
Evaluated the outcomes of your Evidence Based practice	51 (41.1%)	73 (58.9%)	1.780 (1.235–2.564)	0.002	3.448 (1.229–9.671)	0.019*
Share information gathered with colleagues	70 (56.5%)	54 (43.5%)	1.700 (1.166–2.479)	0.006	1.847 (0.637–0.5.356)	0.258
Difficulty in understanding research reports	46 (37.1%)	78 (62.9%)	0.750 (0.570–0.986)	0.039	0.722 (0.525–0.993)	0.045*

*OR odds ratio, CI confidence interval, COR crude odds ratio, AOR adjusted odds ratio*

*Significantly associated with EBM Implementation

## Discussion

The result of this study showed that the Google (75%), Up-To-Date (70.2%) and medical journals (60%) were the main source of evidence for most of the physicians. But the majority of physicians were not aware of or not using electronic databases, mainly HINARI (77.5%), Cochrane (71%), and PubMed/Medline (62%). This finding was lower than a study done in Qatar, 26.4 and 17.0% of physicians were aware of and utilized the Cochrane Database and Best Evidence Review, respectively [2]. Also lower than the study of Oman, and systematic review [3, 22]. It is in line with a study conducted in Egypt (61.3%) physician’s utilized PubMed central [23]. But it is higher than the findings from Black Lion Hospital, Addis Ababa, Ethiopia, Google (57.3%), Cochrane (13%), and HINARI (24%) PubMed (39.5%) and UpToDate (85%) [12]. The difference in finding might be related to the time difference. Currently, for physicians, there is a promotion of EBM implementation by Medical schools, Research institutions, and Nongovernmental organizations who are supporting healthcare.

Correspondingly, the finding from this study in the EBM technical terms shows that Physicians are better understood and explain to others about absolute risk (54%), odds ratio (50.8%), confidence interval, (44.4%), and systematic review (48.4%). This finding is lower than a study conducted in Sudan,the research technical terms known by physicians were risk factors (83%), relative risk (70%), absolute risk (61%), and systematic review (62%) [11]. However, the study is higher than studies conducted in Qassim region, Saudi Arabia; odds ratio (22.9%), relative risk (40.3%), absolute risk (40.6%) and systematic review (31.3%) [24]. The difference in the finding might be related to the difference in the institutional policy and training of physcicians about research.

Physicians have a good attitude (88.7%) towards EBM. The finding is relatively lower than the study conducted at King Abdelaziz University Hospital, Jeddah Saudi Arabia (98.3%) [8] and relatively higher in a study conducted in Yerevan, Armenia (87.60%) [25], Malaysia (85.9%) [26], and Black Lion Hospital, Addis Ababa Ethiopia (60%) [12].

The incorporation of EBM into clinical practice is not sufficient enough to change the quality of health care because only (32.3%) physicians incorporate EBM into their day to day clinical practice. This finding is consistent with the study conducted in Saudi Arabia, (33.40%) [8]. But lower than a study done in polyclinics of Yerevan, Armenia, and Doha, Qatar, (62.30%) [25] and (68.7%) [2]. Nonetheless, it higher than the study conducted in France and Switzerland (14.2%) [27]. The difference might be due to the difference in institutional policy and level of education and specialty, in our case most of the participants are general practitioners.

For the poor integration of the EBM in eastern Ethiopia, different factors and barriers is identified. Among the leading factors, the organization does not have access to free access electronic databases, due to a lack of training in technical terms of research, most of the physicians had difficulty in understanding research reports and do not even know how to find research reports. Furthermore, the Federal Ministry of Health and the hospital’s administration are not committed to implementing the changed practice, including fulfilling necessary resources. In addition, physicians do not have sufficient time and interest to find research reports because of overburdening at work. Finally, the team culture of not welcoming a change in practice is among the leading factors. Similarly, related studies done in Sudan, Armenia, Saudi Arabia, and Ethiopia point out the factors affecting the implementation of EBM into clinical practice are; the availability and access to information; lack of investment by health authorities; lack of free personal time and interest; and lack of skill and lack of facilities [8, 11, 12, 25].

The finding is also consistent with a study conducted in France and systematic review. The most frequent obstacles perceived for the practice of EBM were: lack of general knowledge about EBM, lack of skills for critical appraisal and lack of time [27]. The major barriers to the practice of EBM are related to patient overload and lack of personal time, knowledge, and skills rather than a lack of facilities and resources [3].

### Strength and limitation of the study

The strong point of the survey was that the survey tool was prepared from the formerly used standardized and piloted instrument for measuring Evidence-Based Medicine and its barriers. The study was done on a new area of care and the first study in Eastern Ethiopia so that it can help further studies at the national level to build upon this finding. Since the study design was a cross-sectional study design, it was not possible to establish a temporal relationship between the exposure and outcome variable. The result may not be the representative of the entire physicians in Ethiopia. Finally, the information was obtained through interviewer-administered questionnaire so that response was prone to social desirability bias (the respondents to answer in a manner that will be viewed favorably by others to be seen by interviewers as knowledgeable).

## Conclusion

The attitude of the physicians towards EBM was good but knowledge of EBM, specifically with reference to information sources and technical terms, as well as the practice of integrating new evidence in the healthcare service, was inadequate. This meant that, most physicians continue to provide healthcare services as they have done before without seeing the benefit of incorporating EBM in their practices. This was due to physician-associated factors; poor administration commitment and resource scarcity. To obviate this, the stakeholders need to require a commitment to design a strategy in promoting the practice of EBM. There is a need to increase awareness of, and provide access to, available EBM sources. Formal training of EBM, as well as basic statistical analysis, should be facilitated regularly to nurture an environment favorable to the practice of EBM.

## Data Availability

The datasets used and/or analyzed during the current study are available from the corresponding author on reasonable request.

## Abbreviations

DARE: Database of abstracts of review of effective
EBM: Evidence-Based Medicine
EBP: Evidence-based practice
GP: General practioner
KSA: Kingdom of Saudi Arabia
SPSS: Statistical package for the social sciences
WHO: World Health Organization
